# Long-term efficacy and safety of monotherapy with a single fresh fecal microbiota transplant for recurrent active ulcerative colitis: a prospective randomized pilot study

**DOI:** 10.1186/s12934-021-01513-6

**Published:** 2021-01-19

**Authors:** Haiming Fang, Lian Fu, Xuejun Li, Chunxia Lu, Yuan Su, Kangwei Xiong, Lijiu Zhang

**Affiliations:** 1 Department of Gastroenterology and Hepatology, Second Hospital of Anhui Medical University, Hefei, China; 2 Center for Gut Microbiota Research, Second Hospital of Anhui Medical University, Hefei, China; 3 Department of Gastroenterology, Second Hospital of Anhui University of Chinese Medicine, Hefei, China

**Keywords:** Fecal microbiota transplantation, Inflammatory bowel disease, Ulcerative colitis, Gut microbiota, Intestinal flora

## Abstract

**Background:**

To assess the long-term safety and efficacy of monotherapy with a single fresh fecal microbiota transplant (FMT) for recurrent ulcerative colitis (UC).

**Results:**

Twenty-six eligible patients were enrolled, and 6 patients were excluded. Ultimately, 20 patients were randomized to the FMT group (n = 10) and the control group (n = 10); 80% were females (F/M = 16/4), the mean age was 48 ± 14 years, and the mean duration was 6.4 ± 8.2 years. The mean length of post-FMT follow-up was 19.1 ± 10.1 months (6–38). No statistically significant differences in baseline demographic or clinical characteristics were found between the groups. Ninety percent of patients in the FMT group and 50% of patients in the control group met the primary endpoint at week 8. The Mayo score was significantly decreased compared with that of the control group (n = 10) when reassessed at week 4 (*P* = 0.001) and week 8 (*P* = 0.019) after FMT; there was no significant difference 6 months after treatment. The median remission time was 24 months (95% CI 68.26–131.7%) in both the FMT (range 6–38 months) and control groups (range 7–35 months), with no significant difference (*P* = 0.895). Participants tolerated FMT treatment, and no adverse events occurred during long-term follow-up, with one treatment-related significant adverse event (EBV infection) occurring within 2 weeks after FMT. Stool microbiota composition analysis indicated improved gut microbiota diversity after FMT, with expansion of stool-donor taxa. *Bacteroidetes*, *Firmicutes* and *Proteobacteria* were the dominant bacterial phyla of the gut microbiota in active UC patients. The relative abundance of *Bacteroidetes* decreased and that of *Proteobacteria* increased significantly in active UC patients compared with donors, while *Firmicutes* showed no significant changes. A single fresh FMT could effectively reconstruct the gut microbiota composition in patients with active UC and maintain stability, with increased *Bacteroidetes* and decreased *Proteobacteria* abundance. FMT significantly reduced the relative abundance of *Escherichia* and increased the relative abundance of *Prevotella* at the genus level. Pyruvate metabolism, glyoxylate and dicarboxylate metabolism, and pantothenate and CoA biosynthesis showed significant differences after transplantation.

**Conclusions:**

Monotherapy with a single fresh FMT is an effective and safe strategy to induce long-term remission without drugs in patients with active UC and may be an alternative induction therapy for recurrent UC or even primary UC.

## Introduction

Ulcerative colitis (UC), a major subtype of inflammatory bowel disease (IBD), is characterized by chronic recurrent colorectal mucosal inflammation. The exact etiology and pathogenesis of UC remains to be elucidated. Genetic predisposition, deregulation of immunological responses and intestinal barrier dysfunction, dysbiosis of the gut microbiota and external environmental triggers have been identified as key pathogenic factors for UC. IBD has become a global health burden with increasing incidence and prevalence. Globally, the incidence and prevalence of UC vary in different regions and are highest in Western countries. However, with the Western diet and lifestyle, as well as the widespread use of antibacterial drugs and social pressure, the UC is also increasingly prevalent in newly industrialized countries in Africa, South America and Asia, including China [1–3].

The therapeutic goals for IBD are to achieve long-term induction and maintenance of remission and mucosal healing, reduce complications and improve patients’ quality of life. Post hoc analysis of clinical trials supported the switch of the target from clinical remission to endoscopic healing. Deep remission has been empirically defined as clinical and endoscopic remission [4–6]. Currently, aminosalicylic acid, corticosteroids, immunosuppressants and biological agents are the main drugs used to treat UC. Patients with severe disease or serious complications may even require surgical treatment, including surgery and endoscopic surgery. Although 5-aminosalicylic acids (5-ASAs) are suitable for mild to moderate UC, annual relapse rates of up to 25–40% have been reported despite the use of optimal doses. Corticosteroids play a key role in the induction of remission in active UC but are not suitable for maintenance therapy because there are adverse effects associated with long-term use and a substantial proportion of patients are steroid dependent or steroid resistant. Long-term use of immunosuppressants such as thiopurines can cause serious adverse events. For patients with IBD who have failed traditional therapies, biologics such as infliximab and adalimumab have represented a treatment revolution. However, long-term potential serious adverse events and high costs still limit their clinical application [7–9]. Therefore, new therapeutic strategies still need to be explored.

Fecal microbiota transplantation (FMT) refers to the transfer of gut microbiota from healthy volunteers to patients with gut microbiota dysbiosis-related diseases to restore homeostasis of the gut microbiota, achieving the goal of treatment and even prevention of disease [10]. FMT was first recorded in ancient Chinese physician Ge Hong's “Handbook of Prescriptions for Emergencies” (Eastern Jin Dynasty, AD 317–420). “Drink a liter of healthy fecal juice and you live” was recorded in this famous traditional Chinese medicine work, and this method was mainly used to treat patients with food poisoning or severe diarrhea [11]. Another famous traditional Chinese work titled "Compendium of Materia Medica" (written by Li Shizhen in the Ming Dynasty) also recorded the FMT method. From the perspective of modern medicine, FMT was first reported for the treatment of refractory and recurrent Clostridium infection (RCDI) in 1958 [12]. Currently, FMT is a robust method of manipulating the gut microbiota and an extremely effective approach for RCDI, with an efficacy better than that of vancomycin in randomized controlled trials (RCTs). FMT has been included in the clinical treatment guidelines for RCDI therapy in the United States and European countries [13, 14].

The first case of UC treated with FMT was reported in 1989 [15]. Due to the efficacy and safety of FMT for RCDI, many case reports, case series, and some RCTs have investigated the efficacy and safety of FMT for IBD to date. Our previous research found that FMT is an effective and safe therapy for both pediatric and adult IBD, and it might be a potential rescue therapy and even an initial standardized therapy for IBD [10]. Four RCTs have proven its significant efficacy for inducing remission in active UC [16–19]. UC may represent one of the most robust potential indications for FMT after RCDI [20]. FMT showed its cost-effectiveness, especially on improving the life quality and decreasing the medical and societal cost, for the moderate to severe IBD in a Chinese cohort [21]. However, there are still no standardized guidelines for treating UC with FMT. The indications for FMT, the route of transplantation, the frequency and dose of FMT, donor selection, and donor stool processing remain controversial. More importantly, data on the long-term efficacy and safety of FMT are very limited. The purpose of the present study was to report the long-term efficacy and safety of monotherapy with a single fresh FMT for recurrent active UC. The primary outcome was steroid-free remission of UC, defined as a total Mayo score of 2 with an endoscopic Mayo score of 1 or less at week 8. Secondary clinical outcomes included adverse events, quality of life scores and characteristics of the gut microbiota before and after FMT treatment.

## Results

### Demographic and clinical characteristics

Twenty-six eligible patients who were diagnosed with recurrent active UC were enrolled in this study, and 6 patients were excluded (4 did not meet the inclusion criteria, and 2 declined to participate). Twenty eligible patients with a total Mayo score of 4 to 12 were enrolled and completed the study. The patients were randomized to the FMT monotherapy group (n = 10) and the control group (n = 10). There were no statistically significant differences in baseline demographic or clinical characteristics between the two groups (as shown in Table 1). All enrolled patients had a history of 5-ASA treatment, except one patient with a 5-ASA allergy.

**Table 1:** The baseline characteristics of the study population

Parameters	FMT group	Control group	P value
Total number	10	10	–
Age(year), M ± SD (range)	51.5 ± 12.7 (32–70)	44.6 ± 14.9(22–75)	0.28
Gender, Female/Male	08-Feb	08-Feb	1.000
Disease duration (year)	5.9 ± 7.3 (0.5–25)	6.9 ± 9.3(0.3–30)	0.777
Disease severity			0.895
Severe % (n)	50 (5/10)	40(4/10)	–
Moderate % (n)	40 (4/10)	50(5/10)	–
Mild % (n)	10 (1/10)	10(1/10)	–
Disease extent			1.000
Pancolitis % (n)	50 (5/10)	50 (5/10)	–
Left-sided colitis % (n)	50 (5/10)	50 (5/10)	–
Concomitant medications			–
Mesalazine	90 (9/10)	100 (10/10)	–
Allergy to 5-ASA	10(1/10)	–	–
Total Mayo score	9.5 ± 2.5	8.6 ± 2.9	0.462
Endoscopic Mayo score	2.4 ± 0.7	1.9 ± 0.7	0.137

### Primary outcome at week 8 after treatment

**Fig. 1:**
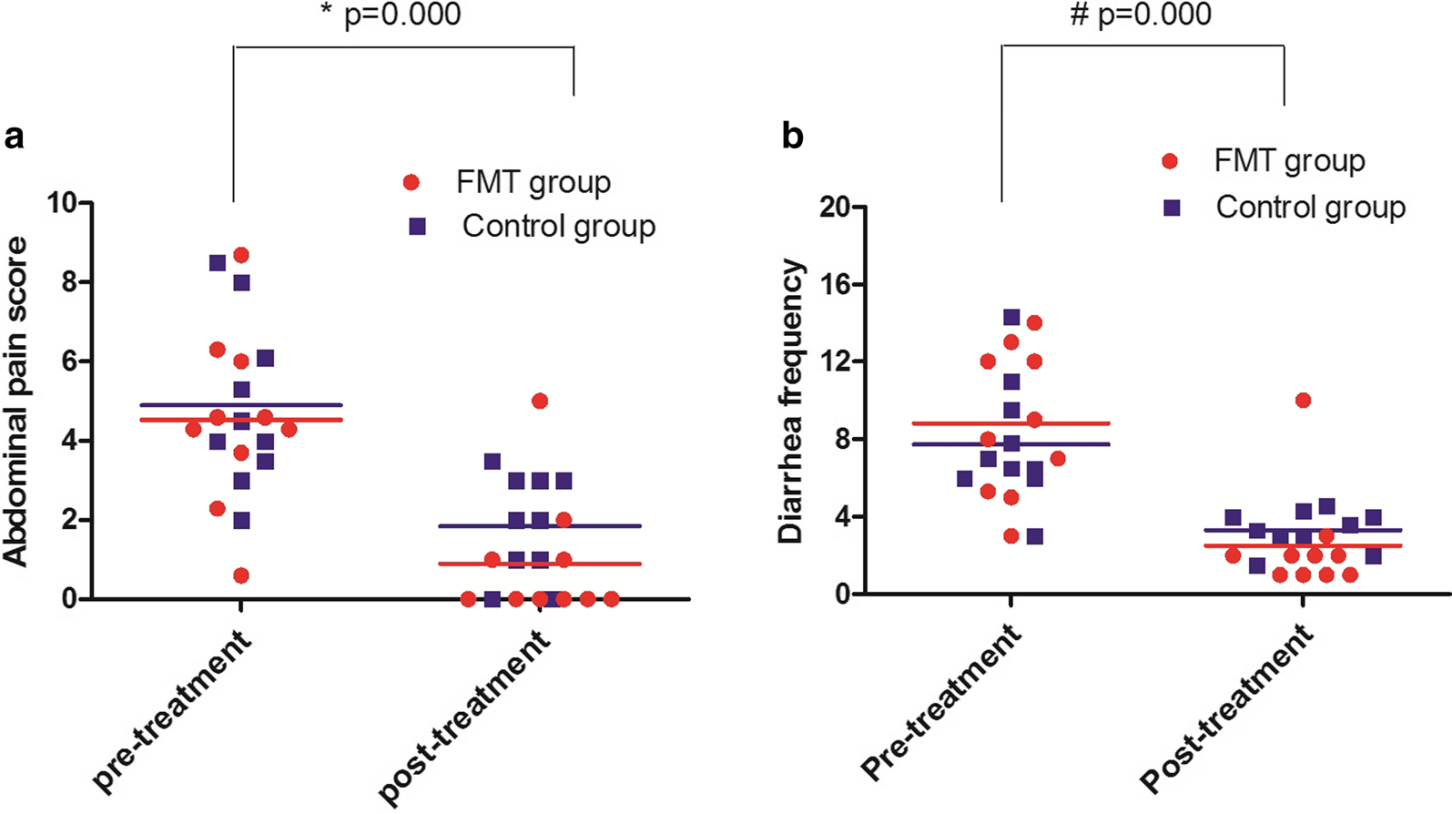
Clinical response to FMT monotherapy (FMT group) and routine therapy (control group) after two weeks of treatment. **a** Abdominal pain score of patients with active UC at baseline and 2 weeks after treatment. In the FMT group, the value was 4.5 ± 2.2 at baseline and 0.9 ± 1.6 after FMT monotherapy (n = 10). In the control group, the values were 4.9 ± 2.1 at baseline and 1.8 ± 1.3 after routine therapy (n = 10). Compared with that observed in pretreatment, abdominal pain significantly improved after FMT treatment and routine therapy (^*^ P = 0.000). **b** Diarrheal frequency of patients with active UC at baseline and 2 weeks after FMT monotherapy. In the FMT group, the value was 8.8 ± 3.8 at baseline and 2.5 ± 2.7 after FMT monotherapy (n = 10). In the control group, the values were 7.8 ± 3.1 at baseline and 3.3 ± 1.0 after routine therapy (n = 10). Compared with that observed in pretreatment, diarrheal frequency significantly improved after FMT treatment and routine therapy (^#^ P = 0.000)

All patients (n = 10) in the FMT group received fresh FMT therapy once, and the mean time point of FMT after donor stool processing was 3.1 ± 0.4 h. Ninety percent (9/10) of UC patients achieved clinical symptom improvement within 2 weeks after FMT, and the mean time point of response after FMT was 22.9 ± 22.5 h. The data are shown in Table 2. Clinical symptoms, including purulent bloody stool, defecation frequency, abdominal pain and abdominal discomfort, were significantly improved. Compared with pretreatment, purulent bloody stool and defecation frequency were significantly decreased. The mean abdominal pain score was significantly decreased from 4.5 ± 2.2 at baseline to 0.9 ± 1.6, and the mean diarrheal frequency was significantly decreased from 8.8 ± 3.8 at baseline to 2.5 ± 2.7 two weeks after FMT Compared with the pretreatment data (abdominal pain and diarrheal frequency), FMT significantly induced clinical remission (P = 0.000) (Fig. 1a, b). In the control group, all patients (n = 10) achieved a clinical response. The mean abdominal pain score was significantly decreased from 4.9 ± 2.1 at baseline to 1.8 ± 1.3, and the mean diarrheal frequency was significantly decreased from 7.8 ± 3.1 at baseline to 3.3 ± 1.0 two weeks after routine therapy (Fig. 1a, b). Fifty percent (5/10) of patients met the primary endpoint assessed at week 8. Compared with that observed in pretreatment, routine therapy also significantly induced clinical remission (P = 0.000).

**Table 2:** Factors related to FMT in patients treated with FMT

Patient	CDI	EBV	CMV	Times of FMT	Time point of FMT after donor stool processing (h)	Time point of response after FMT (h)	Patient-donor relationship	Donor age(yr)
P1	–	–	–	1	2.5	12	Nephew	16
P2	–	–	–	1	3.2	10	Grandson	5
P3	–	–	–	1	2.7	14	Daughter	8
P4	–	–	–	1	3	8	Grandson	13
P5	–	–	–	1	4	12	Grandson	8
P6	–	–	–	1	3	72	Daughter	12
P7	–	–	–	1	3.3	48	Grandson	8
P8	–	–	–	1	2.5	24	Grandson	11
P9	–	–	–	1	2.9	6	Niece	20
P10	–	–	–	1	3	No response	Daughter	12
Mean ± SD	–	–	–	1	3.1 ± 0.4	22.9 ± 22.5	–	11.3 ± 4.4

### Clinical outcomes of long-term follow-up

**Fig. 2:**
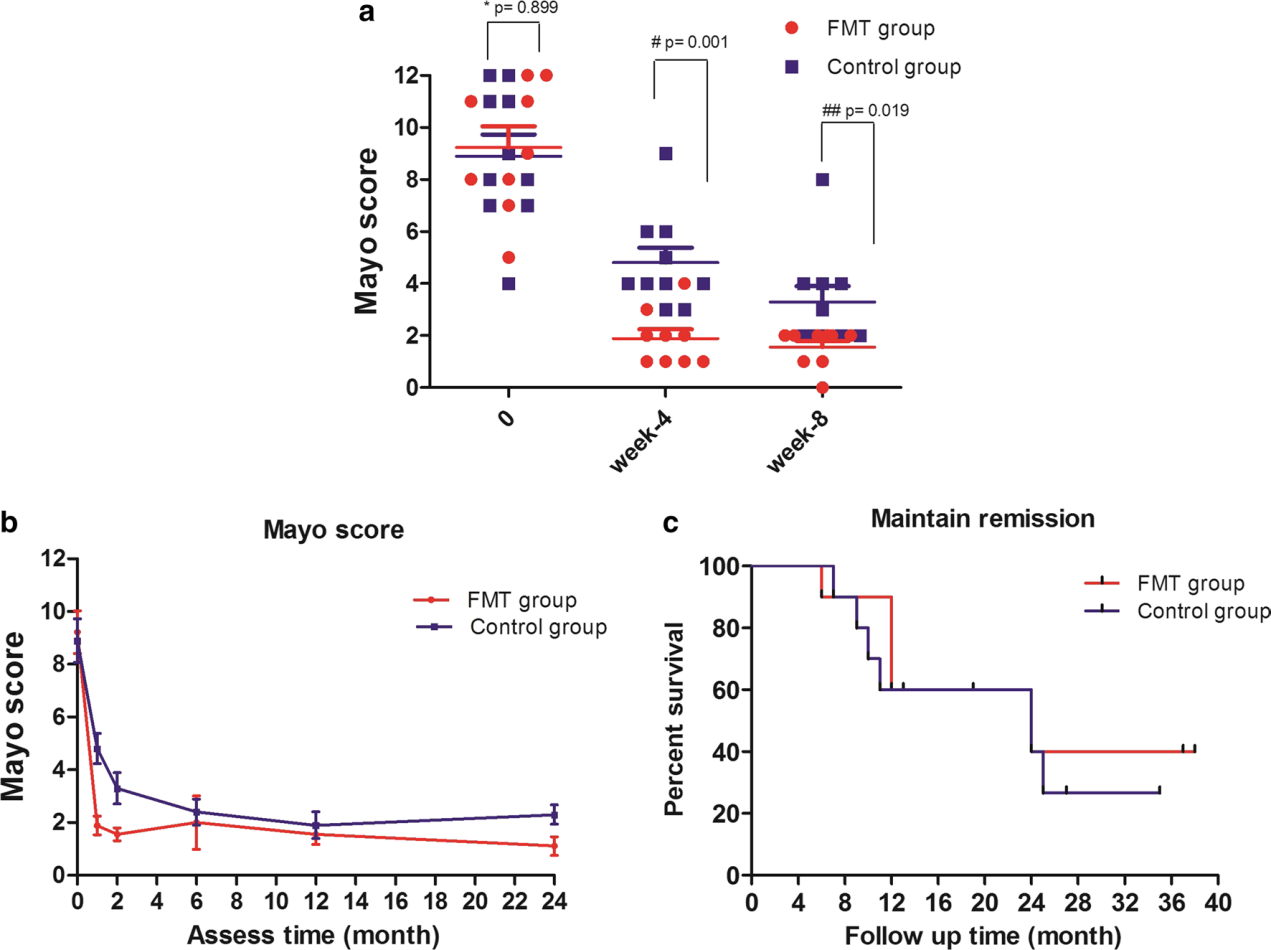
Long-term efficacy of FMT monotherapy or routine therapy for recurrent active UC. **a** Mayo scores at baseline (pretreatment) and post-treatment at week 4 and week 8 in the two groups. Compared with the control group (n = 10), the baseline value showed no significant difference (* *P* = 0.899), while the value was significantly decreased when reassessed at week 4 (# *P* = 0.001) and week 8 (## *P* = 0.019) after FMT treatment (n = 9 FMT responders). **b** Mayo scores after long-term follow-up in the two groups; the scores were not significantly different between the two groups when reassessed after 6 months (*P* = 0.691). **c** Fresh FMT monotherapy resulted in a median of 24 months (range, 6–38) of remission. Routine therapy also achieved a median of 24 months (range, 7–35) remission (95% CI 68.26–131.7%). Monotherapy with a single fresh FMT can achieve long-term remission without drugs in patients with recurrent active UC. There was no significant difference in the maintenance of remission in patients treated with a single FMT compared with the control patients (*P* = 0.895), but patients with active UC who received FMT seemed to achieve clinical remission more quickly

**Fig. 3:**
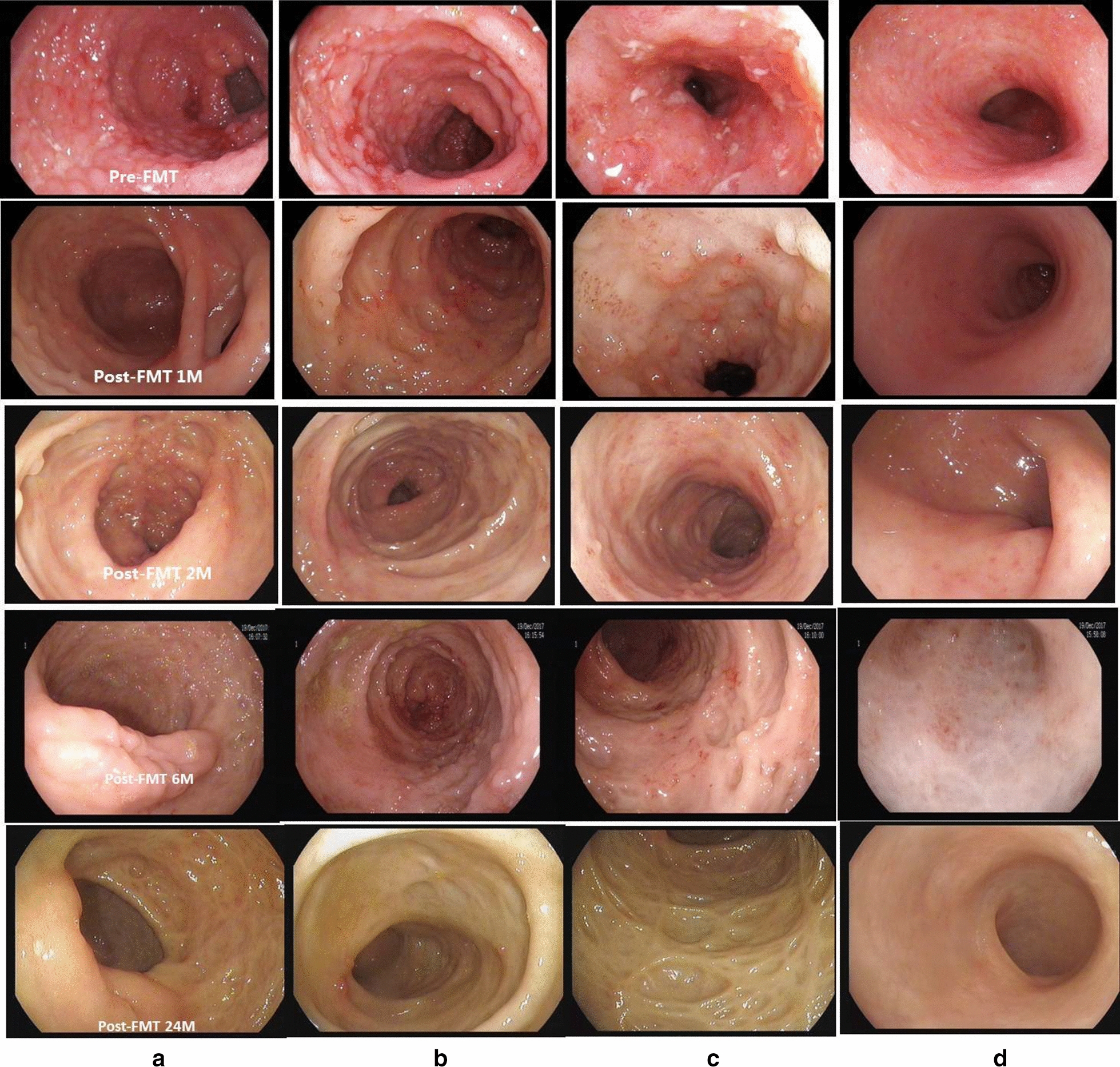
Endoscopic appearance of pancolitis pre- and post-FMT treatment after long-term follow-up in patients with recurrent active UC. The endoscopic appearance includes mucosal hyperemia and edema, mucous exudate erosions, multiple ulcers and spontaneous bleeding in the ileocecum (**a**), ascending colon (**b**), sigmoid colon (**c**) and rectum (**d**) before FMT therapy. Markedly improved lesions with normal mucosa were observed at 24 months after a single fresh FMT. A single fresh FMT can achieve long-term remission without drugs and no obvious adverse events in patients with recurrent active UC

According to the Mayo scoring system, the FMT responders (n = 9) met the primary endpoint at week 8. During the follow-up, one patient who initially responded to FMT maintained remission for 6 months and relapsed. The patient received the same donor FMT treatment via colonoscopy again. Unfortunately, he did not respond to the second FMT treatment and was transferred to corticosteroid induction plus mesalazine maintenance therapy. This patient maintained clinical and mucosal remission during the subsequent long-term follow-up. The remaining 8 FMT responders were reassessed at month 24 after FMT treatment, and five (62.5%, 5/8) patients maintained clinical and mucosal remission with no drugs and no adverse events. During the long-term follow-up, the median maintenance remission time was 24 months (95% CI 68.26–131.7%) in both the FMT group (range 6 to 38 months) and the control group (range 7 to 35 months) (Fig. 2b, c). Endoscopic appearance of pancolitis pre- and post-FMT treatment after long-term follow-up was  shown in Fig. 3.

### Long-term safety of FMT treatment

Adverse events were recorded after FMT and long-term follow-up. All patients could tolerate FMT well, and no serious adverse events occurred. Some patients developed mild abdominal pain, bloating, and nausea, but all of these symptoms resolved spontaneously within 24 h after FMT. One patient developed diarrhea after treatment and was relieved within 24 h without any medical intervention. A 58-year-old female patient achieved clinical remission and was discharged 1 week after FMT. However, she suffered from aggravated purulent bloody stool, defecation frequency abdominal pain and fever two weeks after FMT. The patient was ultimately diagnosed with acute Epstein-Barr virus infection and achieved clinical remission again after antiviral therapy. The patient was able to maintain clinical remission for 24 months with no drugs for UC. However, no evidence of acute Epstein-Barr virus infection was detected in this patient before FMT or in the donor for this patient.

Long-term safety: Although many studies have reported adverse reactions such as fever, abdominal pain, bloating, and diarrhea after FMT, most of these reactions are self-limiting. No side effects were observed, and no infection with certain pathogens was observed during long-term follow-up. No patients suffered from other chronic diseases, such as immune system diseases or nonalcoholic fatty liver disease. All patients had good tolerance to FMT treatment.

### Assessment and analysis of the gut microbiome

**Fig. 4:**
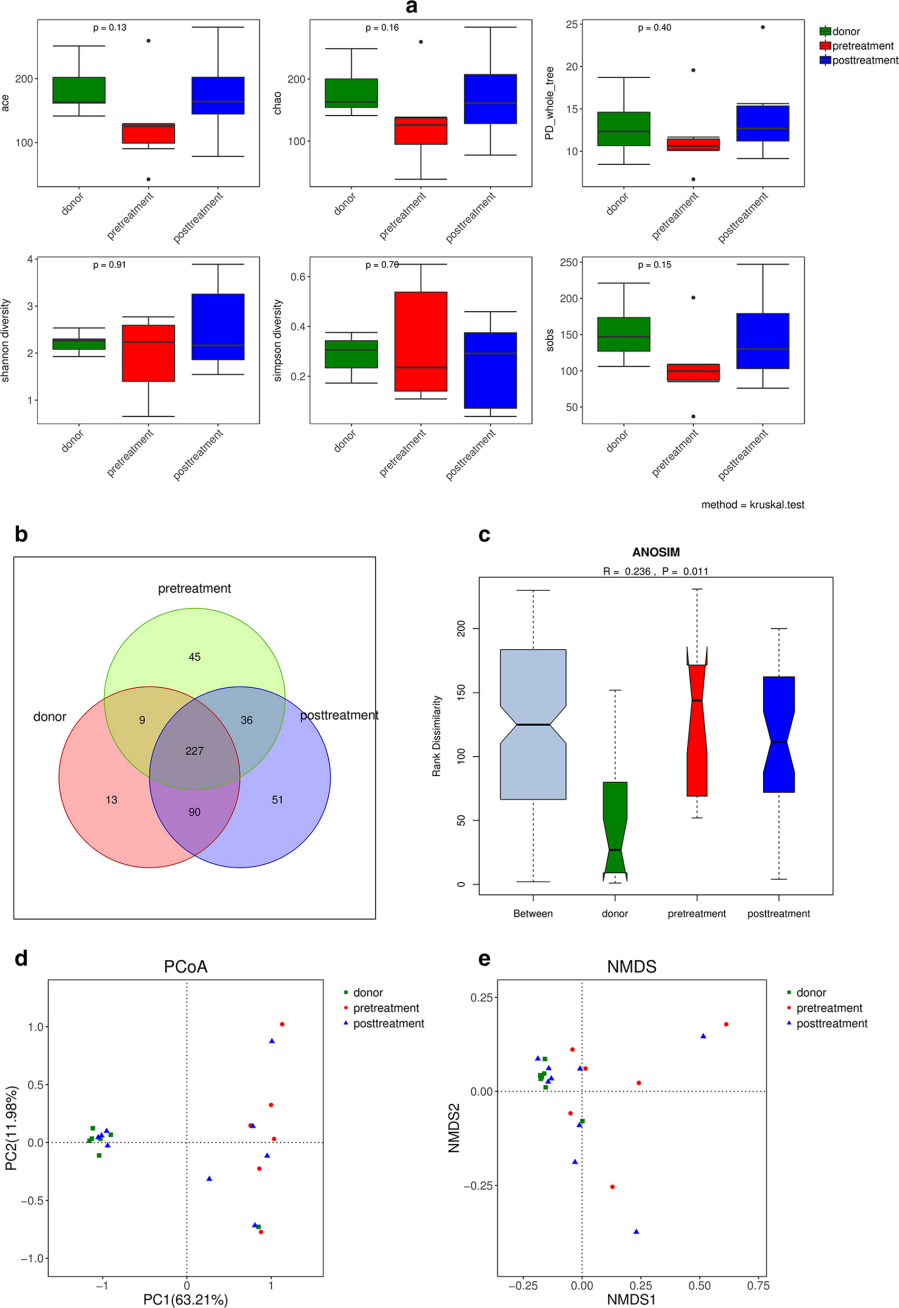
Stool microbiota composition analysis. **a** Alpha diversity index box chart The abscissa represents sample grouping, and the ordinate is the alpha index. The healthy donors, active UC patients (marked as pretreatment) and patients post FMT treatment (marked as posttreatment) showed no significant difference (*p* > 0.05). **b** Venn diagram indicating the number of differential OTUs in each group. **c** Analysis of similarities (ANOSIM) showed significant differences among the healthy donors and pretreatment and posttreatment UC patients. **d** Principal coordinate analysis (PCoA) of the gut microbiota among the healthy donors, active UC patients and patients post FMT treatment. The distance between the samples represents the similarity of the gut microbiota composition, and a closer distance indicates higher similarity. **e** Nonmetric multidimensional scaling plots (NMDS) of the gut microbiota among the healthy donors, active UC patients and patients post-FMT treatment

According to the rarefaction curve plateau of the current sequencing data, most of the diversity has already been captured in all samples. Alpha diversity index calculation was performed with abundance indexes (Chao1 and ACE) and diversity indexes (Shannon and Simpson). The alpha diversity index of the fecal microbiota in active UC patients, healthy donors and patients after FMT treatment showed no significant difference (Fig. 4a, p > 0.05). To measure the level of similarity between gut microbial communities, analysis of similarities (ANOSIM) was performed. The data revealed an apparent separation in the structure of the gut microbiota in each group (Fig. 4c, p = 0.011).

Principal coordinate analysis (PCoA) was used to indicate the similarity of the microbiota composition among samples. PCoA revealed that the gut microbiota in UC patients significantly deviated from that in healthy donors. Treatment with FMT improved the distance markedly, and the samples clustered tightly together, showing a trend similar to that of their related donors, but did not return to the level of healthy donors (Fig. 4d). The system clustering tree also indicated that a significant difference existed between UC patients and healthy donors.

**Fig. 5:**
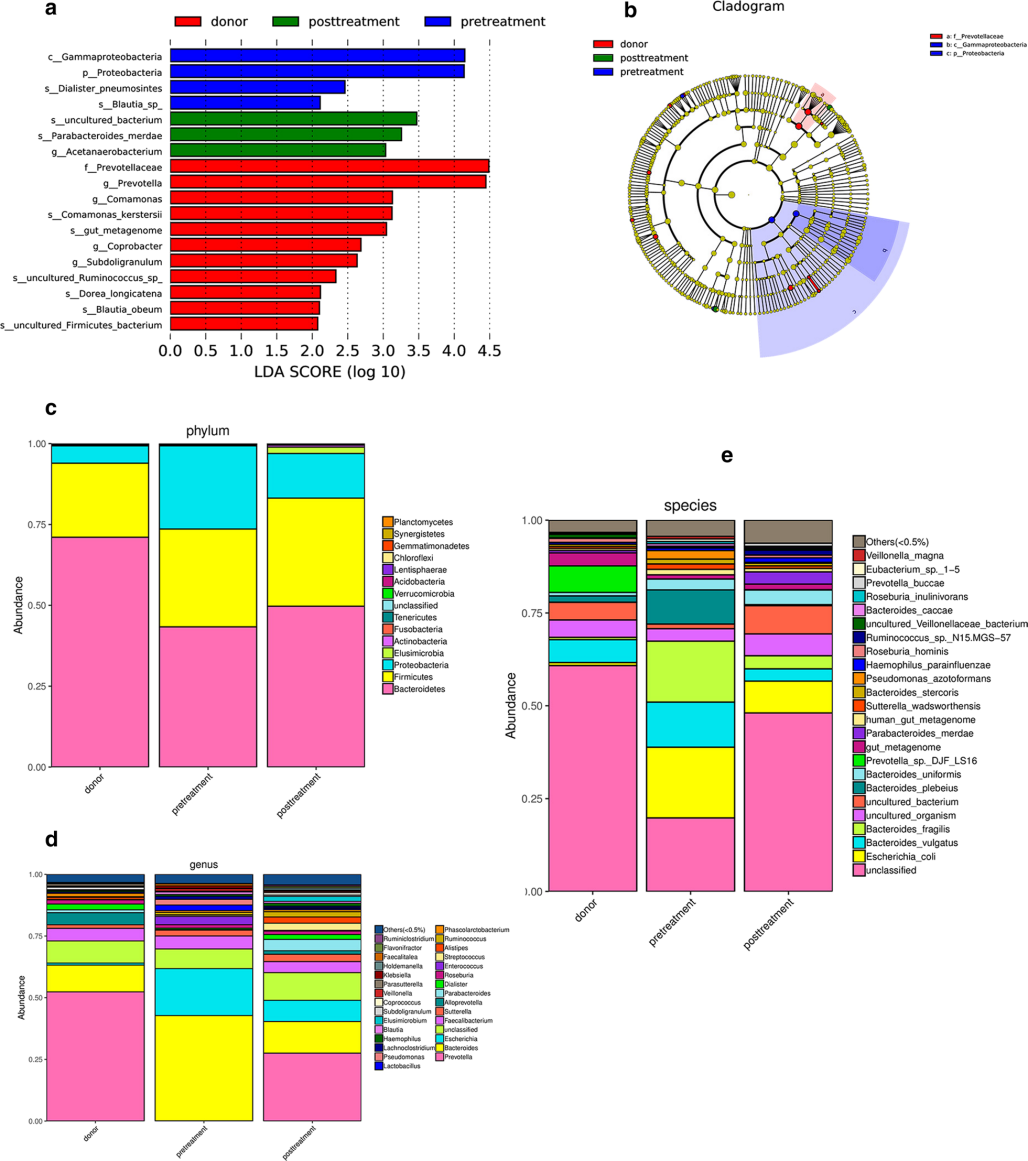
Histogram of taxonomic profiles of the gut microbiota among healthy donors, active UC patients (marked as pretreatment) and patients post-FMT treatment (marked as post-treatment) LDA score (**a**), cladogram (**b**) and profiles at the phylum (**c**), genera (**d**) and species level. *Prevotella* was the dominant genus in the gut microbiota of the healthy donors, and the relative abundance of *Prevotella* increased after FMT treatment in active UC patients

Subsequently, linear discriminant analysis effect size (LEfSe) was used to identify differential microorganism communities between groups. The taxonomic profiles showed that the phyla *Bacteroidetes*, *Firmicutes* and *Proteobacteria* were dominant bacteria in the fecal microbiota of healthy donors and active UC patients. The relative abundance of *Bacteroidetes* was significantly decreased and that of *Proteobacteria* was significantly increased in active UC patients. *Firmicutes* showed no significant changes among healthy donors and active UC patients. Compared with healthy donors, patients with active UC showed an increased ratio of *Firmicutes* and *Bacteroidetes.* Single fresh FMT could significantly reconstruct the dysbiotic gut microbiota and maintain stability, with an increased proportion of *Bacteroidetes* and a decreased proportion *of Proteobacteria*. At the genus level, some specific bacterial biomarkers were identified. The relative abundance of Escherichia was significantly increased in active UC patients and was significantly decreased after FMT. A high abundance of *Prevotella* was found in the donor gut. FMT-treated patients who achieved remission also tended to have a higher abundance of *Prevotella* (Fig. 5).

The PICRUSt tool was used to predict the functional profiles of gut microbiota with the predicted metagenome, and Kyoto Encyclopedia of Genes and Genomes (KEGG) pathway functions were categorized using PICRUSt. PICRUSt predicted analyses found that the gut microbiota pathway functions showed that several pathways in gut microbiome among the donor and pre and post FMT treatment changed significantly, especially the pathways of pyruvate metabolism, sulfur metabolism, pantothenate and CoA biosynthesis, glyoxylate and dicarboxylate metabolism, synthesis and degradation of ketone bodies and other transporters were significantly different between the groups.

## Discussion

FMT is a robust method to increase the diversity of recipient gut microbiota. Engraftment of donor microbiota resulted in a long-lasting response in patients with RCDI [22]. Growing evidence has confirmed that the gut microbiota of patients with UC is characterized by lower diversity and different proportions of certain microorganisms. Manipulation of the gut microbiota via FMT is an emerging novel and promising therapy for IBD [23, 24]. However, due to the complex pathogenesis of UC and multiple influencing factors, the optimum intensity and duration of FMT have not yet been defined.

### Effects of treatment frequency on the outcomes of FMT

One RCT showed that multidonor intensive FMT induced clinical remission and endoscopic improvement in active UC, which was associated with distinct microbial changes that were related to outcome [18]. Another RCT also showed that pooled FMT resulted in a higher likelihood of remission in adult patients with mild to moderate active UC. The diversity of the gut microbiota in patients receiving pooled FMT was greater than that of patients receiving FMT from a single donor, but it was uncertain whether the efficacy was improved [19]. Multisession FMT could induce clinical remission and aid in steroid withdrawal for patients with steroid-dependent UC [25]. For UC patients with remission induced by multisession FMT, a stable dose of standard-of-care treatment (5-ASA with/without azathioprine) plus continuous FMT treatment presented a higher tendency to maintain steroid-free clinical remission and was significantly superior to placebo in terms of both endoscopic and histological remission [26]. Multisession FMT seems to be an effective therapy for maintaining remission in patients with UC. Nevertheless, an open-label pilot study reported that with daily oral multidonor FMT capsules for fifty days as a supplement to standard of care, the symptoms and health-related quality of life in UC patients improved only temporarily [27]. In the present study, 90% of FMT recipients treated with a single fresh FMT achieved clinical symptom improvement within 2 weeks and met the primary endpoint at week 8, particularly in moderately to severely active UC patients; this is consistent with the results of a previously reported meta-analysis [10]. These differences may be correlated with the degree of dysbiosis of the gut microbiota in patients with UC, and it is expected that the inflammatory areas have more severe disease than the noninflammatory areas [28, 29]. The possible effects of other influencing factors, such as the donor and the gut microbiota characteristics of the donors and UC patients, on the efficacy of FMT will also be discussed later.

### Effects of donor selection on the outcomes of FMT

To date, there are no randomized studies comparing efficacy across different protocols; related or unrelated donors should both be considered acceptable, and there are clear advantages to using FMT from a stool bank, from a healthy unrelated donor, particularly with regard to monitoring and traceability [30]. Based on previously reported data, including clinical guidelines, the donors were mostly aged more than 18 years. According to traditional Chinese medicine theory, young healthy volunteers and even children are more suitable than older individuals as donors. One RCT study reported higher treatment success with one particular donor than with the other donors [16]. Donor selection may play a much more important role in the treatment of ulcerative colitis than in the treatment of CDI [31]. In the present study, according to the donor selection criteria, 10 eligible donors (7 female, 3 male) were selected. Their ages ranged from 5 to 20 years old, and the mean age was 11.3 ± 4.4 years old. Every donor was a direct relative of the FMT receptor. The UC patients responded well to monotherapy with a single fresh FMT, which may be related to the selection of young donors. Recently, a cross-sectional study revealed that sex differences in the gut microbiota composition and predicted metabolic profiles exist before puberty, which become more significant at puberty [32]. Gender and age may play an important role in FMT donor selection, and even the clinical efficacy of FMT. This may be an interesting topic for further study, especially in RCTs.

### Roles of gut microbiota reconstruction via FMT

Through assessment and analysis of the gut microbiome in UC and healthy donors, we found that the diversity and richness of the gut microbiota of active UC patients were significantly different from those of healthy donors. The diversity was reduced, the relative abundance of *Bacteroides* was decreased, and that of *Proteobacteria* was increased significantly, but *Firmicutes* showed no significant change. The dysbiotic gut microbiota in active UC patients may be reconstructed by FMT, resulting in a microbiota similar to that of the donor. At the genus level, the relative abundance of *Escherichia* decreased, and the level of *Prevotella* markedly increased after FMT. Phyla *Bacteroidetes* is mainly composed of the genera *Bacteroides* and *Prevotella*, which are thought to share a common ancestor. *Bacteroides* is the predominant bacteria in the human gut among people with a Western diet characterized by high protein and animal fat contents; in contrast, in non-Westernized populations consuming a plant-rich diet, *Prevotella* dominates in the gut microbiota [33]. *Prevotella* is a large genus with high species diversity and high genetic diversity among strains, which makes it difficult to predict their function with obvious individual differences [34]. The role of *Prevotella* in the pathogenesis of UC is still controversial. One single species isolate, *Prevotella copri* CB7, has been used for different studies and can be beneficial or detrimental, depending on the context [34]. Intriguingly, some papers suggested that *Prevotella* might be regarded as a beneficial bacterium but not a pathobiont. For example, the enrichment of *Prevotella copri* was observed in healthy individuals taking a fiber-rich diet that normally exhibits anti-inflammatory activities [34, 35]. The most consistent finding is that *Escherichia*, specifically *Escherichia coli,* was increased in IBD. However, due to technical limitations, the vast majority of microbiota studies in IBD have not analyzed the microbiota at the strain level. Therefore, it is unknown whether the increase or decrease in certain microbiota is due to pathogenic strains or “protective” strains [36]. An individual’s response to FMT may predominantly depend on the capability of the donor’s microbiota to engraft and reverse the microbial community dysbiosis associated with a specific disease phenotype; these factors need to be investigated in future studies [37].

### Long-term efficacy and safety of FMT monotherapy in patients with active UC

Borody et al. [38] reported that 6 patients with UC received an FMT enema for five consecutive days, and all patients achieved complete reversal of symptoms and maintained remission for 1–13 years with no drugs required. Ding [39] reported that after a follow-up of 1–5 years, step-up FMT was safe and effective for patients with moderate to severe UC and improved the quality of life. However, this step-up FMT strategy involves repeated FMT; step 1 was FMT monotherapy and step 2 was multiple FMTs (at least 2), followed by one or more FMTs in combination with corticosteroids or cyclosporine if there was no response to step 1 or 2. In our study, monotherapy with a single fresh FMT can achieve long-term remission without drugs and no obvious adverse events in patients with recurrent active UC. Compared with the control group, patients treated with a single FMT showed no significant difference in maintenance of remission, but patients with active UC who received FMT seemed to achieve clinical remission more quickly. We speculate that various factors could contribute to the positive outcomes achieved in our study. First, the fresh fecal slurry contains more beneficial microorganisms and metabolites. The fresh transplant strategy within 6 h reduced the loss of beneficial microorganisms. Furthermore, the colorectal instillation of fresh fecal slurry using the colonoscopic route ensured good volumes of donor fecal slurry. All FMT recipients were required to remain in bed for at least for 60 min without defecation, which ensured a good retention time. The role of bowel preparation in the published literature is controversial. Some views hold that adequate pre-FMT bowel preparation may help clear proinflammatory bacteria and allow donor microbiota to colonize the recipient gut [40]. However, some believe that bowel preparation with polyethylene glycol may itself cause changes in the microbiota, thus producing results not attributable to FMT [41]. FMT may contribute towards disease remission in UC, but the factors determine long-term effect of treatment is not completely clear. Matching between donors and UC patients is important for long-term maintenance after FMT [42]. Sustained remission was associated with known butyrate producers and overall increased butyrate production capacity, while relapse was associated with *Proteobacteria* and *Bacteroidetes* [43]. Of course, the present research is a small sample study, and the efficiency may change or even decrease with the increase of the sample size. Thus, large-sample RCTs are needed.

### Summary

Single fresh FMT is an effective and safe strategy to induce long-term remission in patients with active UC. Single fresh FMT could effectively reconstruct the composition of the gut microbiota in patients with active UC. FMT is expected to be an alternative induction therapy for recurrent UC and even primary UC. Although this study had a small sample, it could still provide a reference for treating active UC, especially recurrent UC, due to the long-term follow-up.

## Materials and methods

### Patients and study design

Patients with recurrent active UC who were hospitalized in the Department of Gastroenterology and Hepatology, Second Hospital of Anhui Medical University, from April 2017 were enrolled in this study. The diagnosis of patients with recurrent active UC depended on the typical clinical symptoms, endoscopic assessment and histological findings. The Mayo scoring system was used to evaluate disease extent. Subjects with a Mayo score of 4 to 12 who had previously received 5-ASA treatment at a stable dose for at least 4 weeks but had received no other therapy, including immunosuppressive agents, biologics and surgery, were included. Patients with UC who were pregnant, had a history of abdominal surgery or FMT treatment, and had been exposed to antibiotics, probiotics or prebiotics in the prior 4 weeks were excluded. Evidence of other infections, such as *Clostridium difficile*, cytomegalovirus, Epstein-Barr virus or extraintestinal infections requiring antibiotics, was excluded. Patients with other comorbidities, such as heart, lung, and cerebrovascular disease, history of gastrointestinal malignancy, polyps or IBS, abdominal surgery, or inability to undergo endoscopy, were also excluded.

The study was designed as an open-label, randomized, parallel-group comparison study. Eligible patients were randomized to the FMT monotherapy and control groups. Subjects in the FMT group were treated with monotherapy with a single fresh FMT via colonoscopy. Subjects in the control group received routine therapy as follows: patients with mild to moderate UC were treated with mesalazine, and patients with severe UC were subsequently treated with corticosteroids for induction therapy and mesalazine for maintenance therapy. Eligible participants were aged 18–75 years and of either sex. All patients provided written informed consent. The ethics committee of the Second Hospital of Anhui Medical University approved the protocols.

### Donor selection and donor stool processing

Potential healthy stool donors were found via a strict screening questionnaire, a subsequent medical interview, and an examination followed by blood and stool testing to minimize the risks of disease transmission, as previously described [26]. The healthy stool donors had no personal or family history of irritable bowel syndrome, chronic constipation, chronic diarrhea, gastrointestinal cancer, polyps, intestinal tuberculosis or any other chronic gastrointestinal diseases. There was no personal or family history of diabetes, metabolic syndrome, obesity, hypertension, malnutrition, liver/kidney dysfunction or any other autoimmune or allergic disease, such as eczema or asthma. No commonly detectable enteric pathogens, such as *Entamoeba coli, Clostridium difficile, or tuberculosis*, were detected by stool microscopy and culture. No evidence of infectious diseases, such as Epstein-Barr virus, cytomegalovirus, hepatitis A, B, C, D or E virus, syphilis, and human immunodeficiency virus (HIV), was found by blood testing. There was no history of drug abuse or recent gastrointestinal surgery and no history of antibiotics, chemotherapy drugs, or immunosuppressive agents in the last 3 months. Written informed consent was obtained from all donors or their guardians.

Fresh feces were processed in the morning on the day of FMT. Fresh donor feces were processed within 1 h after the donor defecated. In brief, 50 g of freshly passed donor feces was dissolved in 250 ml of sterile 0.9% physiological saline for 5 min with a conventional blender and then sequentially filtered through 2.0 mm, 1.0 mm, 0.5 mm, and 0.25 mm stainless steel filters. Finally, the filtered liquid was centrifuged (6000 r/min) at 4 °C for 15 min, and the precipitate was redissolved in 150 ml of sterile physiological saline for FMT by colonoscopy.

### FMT procedure

Patients in the FMT group were not allowed to take antibiotics or aminosalicylic acids such as sulfasalazine or mesalamine, and a light diet was recommended 2–3 days before FMT. The participants received bowel lavage (polyethylene glycol electrolyte dissolved in 2 L of water, an isotonic whole-intestine lavage fluid composed of 125 mmol/L sodium ions, 10 mmol/L potassium ions, 20 mmol/L bicarbonate ions, 40 mmol/L sulfate ions and 35 mmol/L chloride ions) 4–6 h prior to FMT. Fresh FMT was performed within 4–6 h after the donor feces were processed. In detail, a total of 200 mL of donor fecal slurry was delivered into the right and left colon via colonoscopy. Upon completion of the transplant, all recipients were required to remain in bed for at least 60 min without defecation.

### Clinical outcomes and follow-up

The primary endpoint was clinical and mucosal remission at week 8. Clinical remission was defined as a Mayo score ≤ 2 with each subscore ≤ 1, and mucosal remission was defined as a Mayo endoscopy subscore ≤ 1 compared with baseline. Clinical response was defined as a decrease in Mayo score of ≥ 30% and ≥ 3 points when compared with baseline at week 8. A subitem score for bloody purulent stool that decreased ≥ 1 score or a score of 0 or 1 was defined as clinical improvement. Patients who achieved a clinical response were also enrolled in the clinical response analysis.

The secondary endpoints were the maintenance of clinical and mucosal remission and possible adverse events during long-term follow-up. Patients were followed up at weeks 2, 4, 8, 12, and 24 and months 12 to 24 after treatment. Colonoscopy findings and Mayo scores were recorded at week 0 (baseline) and at weeks 4, 8, 24 and months 12 and 24 after treatment.

Clinical relapse was defined as exacerbation of diarrhea and purulent bloody stool that required drugs, including initiation or replacement of drugs, to induce remission. All patients were followed up by telephone or outpatient service.

### Assessment and analysis of the fecal microbiota by 16S rRNA sequencing

Fresh fecal samples from the donors and pre-FMT and post-FMT samples from patients were collected using a sterile collection spoon and stored in 3 ml of preservation solution at − 80 °C for analysis. The gut microbiota was assessed by 16S ribosomal RNA sequencing. The V3–V4 hypervariable region of the 16S rRNA gene was amplified via high-throughput sequencing on the Illumina MiSeq platform, and the raw sequencing data from stool samples from individual donors and FMT recipients pre- and post-FMT treatment were processed into operational taxonomic units at 97% similarity.

### Statistical analyses and visualization

An intention-to-treat analysis was performed. Baseline demographic, medication and disease parameters, including disease duration, severity and extent, are presented using means (SDs) or frequencies (percentages). The categorical variables between groups were compared by the Chi-square test. The clinical response in both groups was compared by using Student’s t test. SPSS Statistics v17.0 was used for statistical analysis. P < 0.05 was considered statistically significant.

In a previous study [44] estimates of alpha diversity were based on an evenly rarefied OTU abundance matrix and included observed richness, observed species, and the Shannon, Simpson, ACE, and Chao1 indexes using the R package vegan. The significance of differences in measured alpha-diversity metrics across samples was tested using a nonparametric Kruskal–Wallis rank sum test and the Benjamini–Hochberg correction. The beta diversity of the samples was measured using the Bray-Curis distance based on an evenly rarefied OTU abundance table. The β-diversity can estimate the difference in community structure between samples. Statistical differences in measured β-diversity metrics across groups were determined using PERMANOVA with 999 permutations, using adonis in the R package vegan. Shared OTUs were calculated and visualized using the R package Venn diagram. The taxon abundance was measured and plotted using ggplot2. LEfSe analysis was performed to identify taxa with differential abundance in the different groups. LEfSe is an algorithm for high-dimensional biomarker discovery and explanation that identifies genomic features that characterize the differences between two or more biological conditions. Moreover, indicator analysis based on genera was conducted using the R package. Indicator taxon analysis was a way to calculate the probability that any taxon was found in different groups; a taxon with a high indicator value has a high probability of being found within a given treatment and a low probability of being found outside the treatment, and the p-values were corrected with the method of Benjamini–Hochberg using p.adjust in R. Finally, the results were visualized using a custom R script based on ggplot2. These analyses were performed using R v3.4.1.

To predict metagenomic functional content, Phylogenetic Investigation of Communities by Reconstruction of Unobserved States (PICRUSt) was used to predict which genes are present using 16S data. The software utilizes a computational approach to predict functional pathways from 16S rDNA reads. First, the reads were searched against a reference collection, Green Genes database, May 2013 version, and the closed-reference OTU table was built using QIIME. The resulting OTU table was normalized by normalize_by_copy_number.py. Metagenome predictions were conducted using predicted metagenomes.py. The significant difference analysis was performed using ANOVA. The results were visualized using a custom R script based on ggplot2.

## Data Availability

The datasets generated and/or analysed during the current study are available in the [PRJNA659913] repository, [https://submit.ncbi.nlm.nih.gov/subs/bioproject/].

## Abbreviations

UC: Ulcerative colitis
IBD: Inflammatory bowel disease
FMT: Fecal microbiota transplantation
RCDI: Recurrent Clostridium infection
RCTs: Randomized controlled trials
OTU: Operational taxonomic unit
ANOSIM: Analysis of similarities
PCoA: Principal coordinate analysis
NMDS: Nonmetric multidimensional scaling plots
LEfSe: Linear discriminant analysis (LDA) effect size
